# Intraspecific variation in male mating strategies in an African ground squirrel (*Xerus inauris*)

**DOI:** 10.1002/ece3.9208

**Published:** 2022-08-15

**Authors:** Mary Beth Manjerovic, Eric A. Hoffman, Christopher L. Parkinson, Jane M. Waterman

**Affiliations:** ^1^ Department of Biology Virginia Military Institute Lexington Virginia USA; ^2^ Department of Biology University of Central Florida Orlando Florida USA; ^3^ Department of Biological Sciences Clemson University Clemson South Carolina USA; ^4^ Department of Biological Sciences University of Manitoba Winnipeg Manitoba Canada; ^5^ Department of Zoology and Entomology Mammal Research Institute, University of Pretoria Pretoria South Africa

**Keywords:** competition, mating, resources, Sciuridae, *Xerus inauris*

## Abstract

Male mating strategies respond to female availability such that variation in resources that affect spatial distribution can also alter cost–benefit tradeoffs within a population. In arid‐adapted species, rainfall alters reproduction, behavior, morphology, and population density such that populations differing in resource availability may also differ in successful reproductive strategies. Here, we compare two populations of Cape ground squirrels (*Xerus inauris*), a sub‐Saharan species with year‐round breeding and intense mating competition. Unlike most mammals where males resort to aggressive interactions over females, male *X. inauris* are tolerant of one another, relying instead on other nonaggressive pre‐ and postcopulatory strategies to determine reproductive success. Our findings suggest that differences in resource availability affect female distribution, which ultimately leads to intraspecific variation in male reproductive tactics and sexual morphology. Sperm competition, assessed by reproductive morphometrics, was more pronounced in our high resource site where females were distributed evenly across the landscape, whereas dominance seemed to be an important determinant of success in our low resource site where females were more aggregated. Both sites had similar mating intensities, and most males did not sire any offspring. However, our low resource site had a higher variance in fertilization success with fewer males siring multiple offspring compared with our high resource site where more individuals were successful. Our results lend support to resource models where variations in female spatial distribution attributed to environmental resources ultimately impact male reproductive behaviors and morphology.

## Graphical abstract

Male mating strategies respond to female availability such that variation in resources that affect spatial distribution can also alter cost benefit tradeoffs within a population. We compared two populations of Cape ground squirrels (*Xerus inauris*), an arid‐adapted species with year‐round breeding and intense mating competition, where males are rarely aggressive towards one another and rely on pre‐ and post‐copulatory strategies to determine reproductive success. Our findings suggest the differences in resource availability and female distribution lead to intraspecific variation in male reproductive behavior and morphology.

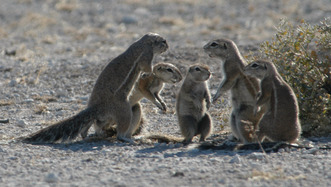

## 1 INTRODUCTION

Many aspects of species' ecology and behavior, including social and reproductive strategies, are strongly influenced by environmental conditions (Clutton‐Brock & Harvey, 1978; Millán et al., 2021; Schradin et al., 2017). Resource‐based models suggest ecological parameters impact the social organization as females respond to the distribution and quality of resources and environmental risks (Emlen & Oring, 1977; Maher & Burger, 2011; Rémy et al., 2013). The spatial and temporal distribution and density of receptive females impact competition dynamics by altering the costs and benefits of acquiring partners and ultimately influence male reproductive decisions and physiology (Brashares & Arcese, 2002; Emlen & Oring, 1977; He et al., 2019; Schradin et al., 2010; Shuster & Wade, 2003). In species lacking male parental care, male reproductive success is generally limited by the number of acquired mates, such that males balance current and future reproductive opportunities depending on whether females are economically defendable in space and time (Clutton‐Brock, 1989;Emlen & Oring, 1977; Schwanz et al., 2016). When females are dispersed or when reproduction is asynchronous, traits that enhance male mate searching abilities and dominance hierarchies will be favored (Emlen & Oring, 1977; Schwanz et al., 2016). Alternatively, females clumped together with discrete breeding seasons favor traits that aid males in direct competition or monopolization (Lacey & Wieczorek, 2001; Waterman, 2007).

In promiscuous species, postcopulatory competition also explains many aspects of variation in male and female reproductive traits (Córdoba‐Aguilara, 2005; Minder et al., 2005) and mating behaviors (DelBarco‐Trillo & Ferkin, 2006; Dixson, 2021). Competition accounts for variation in sperm physiology and morphology (Dixson & Anderson, 2004; Gomendio et al., 2006), and male reproductive anatomy (i.e., testes and accessory glands) often is used as a proxy for the intensity of sperm competition (Dixson, 2021; Ramm et al., 2005). However, these reproductive traits are physiologically expensive and thus can be directly impacted by resources. In the Arabian spiny mouse (*Acomys dimidiatus*), rainfall increased follicle counts and gonad (i.e., testes and ovaries) mass and volume, and altered hormone profiles for both sexes (Sarli et al., 2016). As is generally the case in desert environments where there are unpredictable changes in water and/or food availability, this species maintains reproductive function throughout the year to ensure rapid responses to reproductive opportunities as they arise (Bronson, 2009; Sarli et al., 2016).

**FIGURE 1:**
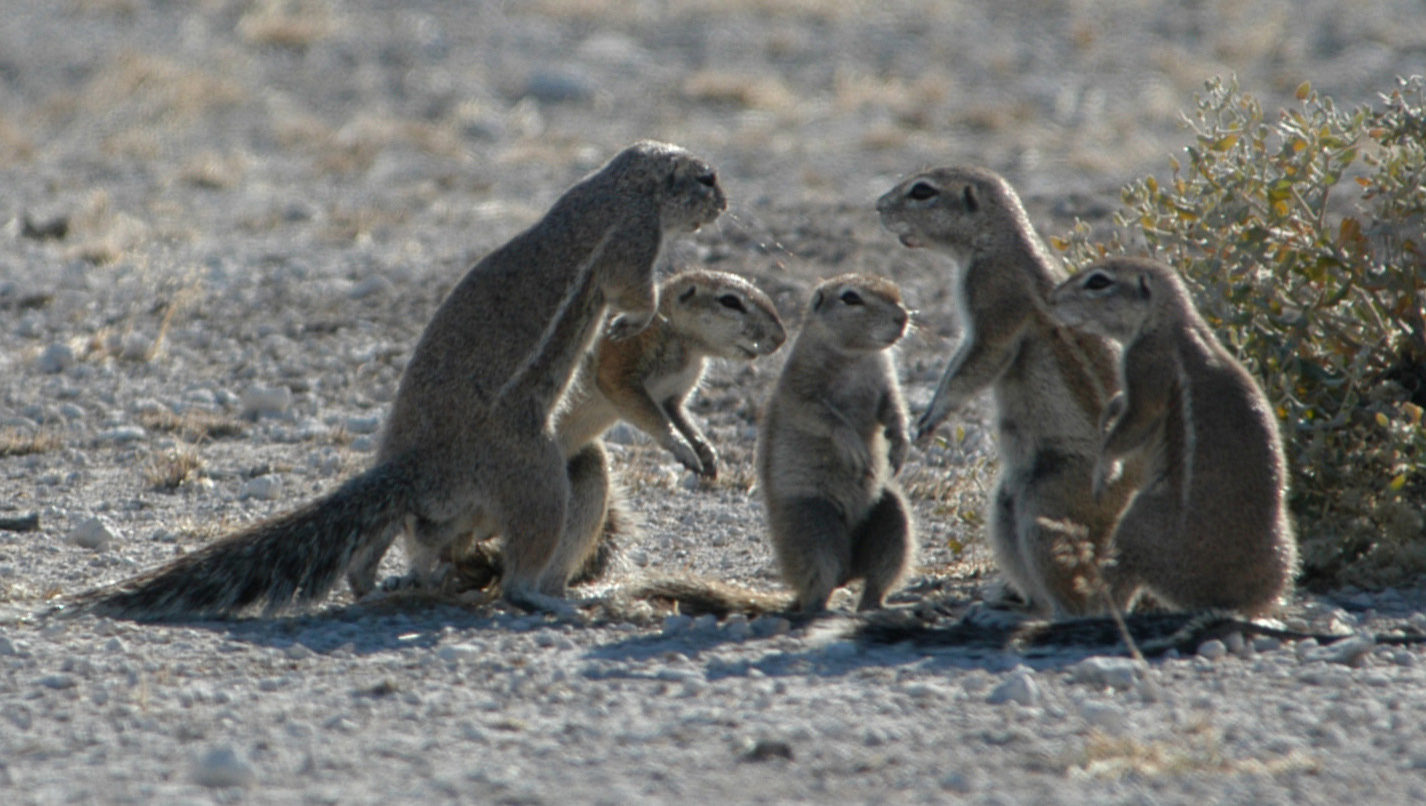
Male Cape ground squirrels (*Xerus inauris*) of different ages often associate with each other in non‐aggressive interactions

Ground‐dwelling sciurids represent a continuum of social organization and mating systems, making them an ideal clade to explore variation in male reproductive strategies (Schwanz et al., 2016). The Cape ground squirrel (*Xerus inauris*) lies at one end of that spectrum with extreme promiscuity and sociality (Figure 1). They differ from other social species by forming year‐round, unrelated, all‐male groups independent of females (Waterman, 1995; Waterman, 1997). Male coalitions often are products of various ecological and demographic parameters such as habitat type, dominance hierarchy, or estrous duration (Díaz‐Muñoz et al., 2014; Olson & Blumstein, 2009; Waterman, 1997), but they are rarely given the paradox of males competing for reproductive opportunities (Clutton‐Brock, 2009; Trivers, 1972). In most polygynous species, intense sexual selection leads to male–male conflict ultimately driving male‐biased mortality. However, little is known about life‐history tradeoffs and mating in species where males lack overt conflict (Bonduriansky et al., 2008). Rather than aggressively competing to defend females or maintain territories, male *X. inauris* move throughout the landscape searching for estrous females in the presence of other unrelated competitors (Waterman, 1995, 1997, 1998). Mate order is determined by a linear dominance hierarchy maintained by nonaggressive displacements (Waterman, 1995, 1998). Males compete with other males via precopulatory competitive searching (Waterman, 1995, 1998) and sperm competition (Manjerovic et al., 2008). Litter sizes of one to two suggest the likelihood of multiple paternity is low, although females average 4 mates (Waterman, 1996, 1998), and multiple paternity is possible (Manjerovic & Waterman, 2015).

Here, we present data from two populations of *X. inauris* to examine how competition can vary within a species that lacks overt conflict. The sites are known to belong to the same phylogenetic clade (Herron et al., 2004) and do not vary in female reproductive output (Pettitt et al., 2008). However, the sites differ in rainfall and resource availability (LaFlèche & Waterman, 2020; Pettitt et al., 2008; Waterman, 1995), setting up a natural comparison to address how resources may influence reproductive competition among males. Male and female social groups live apart in complex, underground burrow systems or clusters, that consist of multiple burrow openings separated from adjacent clusters by areas without burrows (Herzig‐Straschil, 1978; Waterman, 1995). We predict that as female family groups become more aggregated, male home ranges should shrink and males should be more likely to monopolize mates based on a dominance hierarchy, increasing precopulatory reproductive competition. However, as females become more evenly distributed across the landscape, monopolizing females becomes less advantageous, resulting in males competitively searching for females and increasing selection for postcopulatory strategies including sperm competition. Ultimately, as competition for mating opportunities intensifies, we predict greater variance in overall reproductive success among males.

## 2 METHODS

### 2.1 Site variation

We collected field data between 2002 and 2006 at two sites with known differences in both quantity and variability of rainfall (O'Brien et al., 2021; Pettitt et al., 2008) and presumably, resource availability as there tends to be a strong correlation between rainfall and primary productivity in arid and semi‐arid environments (Happold & Happold, 1992). Our “high resource site,” located in central South Africa (27°35′S, 25°35′E), receives an average of 502 mm annual rainfall and has a contiguous distribution of *Eragrostis* spp. of grasses (LaFlèche & Waterman, 2020; Pettitt et al., 2008; van Zyl, 1965). Our “low resource site,” located in east‐central Namibia (23°25′S, 18°00′E), averages 220 mm annual rainfall (LaFlèche & Waterman, 2020; Waterman, 1995) and is predominantly *Acacia* bush with patchy distributions of grasses dominated by *Schmidtia kalahariensis* (Waterman, 1995). Ground squirrels have been previously studied at this low resource site from 1989 to 1991 (Waterman, 1995, 1996, 1998) and during the current study (2002–2006). While we know average total rainfall differs between sites (LaFlèche & Waterman, 2020; Pettitt et al., 2008), we also looked at the variance in rainfall between sites using a Levene's test with data collected at each site from 1980 to 2006.

To determine whether differences in rainfall reflect resource availability, we quantified percent cover at a subset of burrow clusters (*n* = 6) at each site in 2005 and 2006. We estimated percent cover at the same locations each year, collecting data at each site within 1 month of each other, prior to the rainy season, using a 1 × 1 m quadrat spaced north and south at 10, 50, 100, and 150 m from the center of the cluster. We tested for differences between sites and years using a two‐way anova. To address the distribution of suitable habitats between sites, in 2006, we plotted all known burrow clusters in ArcMap v.9.3.1 (ESRI). Female social groups live in the same burrow cluster for multiple years, actively maintaining them such that burrow clusters rarely change between years (Ewacha et al., 2016; Herzig‐Straschil, 1978). We used locations of individual burrows in a given burrow cluster to generate 95% minimum convex polygons (MCP) and calculated the total area of known burrow clusters and the average distance between burrow clusters. We used the multi‐distance spatial cluster analysis to calculate the dispersion of burrow clusters based on Ripley's K (Ripley, 1976). This analysis generates an expected pattern of complete spatial randomness compared with the observed burrow distances as an indication of clustering or dispersion (Wilschut et al., 2015).

### 2.2 Population sampling

We trapped squirrels using Tomahawk© live traps (Tomahawk Live Trap Co., Hazelhurst, WI; 15 × 15 × 50 cm) baited with peanut butter and bird seed following methods outlined in Waterman (1995). Upon capture, we transferred animals into a handling bag to reduce stress (Koprowski, 2002). We recorded standard measurements including body mass (measured with a spring scale to ±5.0 g), sex, reproductive condition, and age; individuals were uniquely marked for short‐and long‐term identification using hair dye and pit tags (Hillegass et al., 2008; Waterman, 1995). We collected a 1‐ to 3‐mm sample of tail tissue for genetic analysis and stored samples in 95% EtOH at room temperature (Manjerovic & Waterman, 2015). After handling, we released all animals at the site of capture. Handling was in accordance with the American Mammal Association guidelines (Gannon & Sikes, 2007) and was approved by the University of Central Florida's IACUC (#07‐43W).

### 2.3 Demographics

We used trapping and observation data collected between 2002 and 2006 to determine yearly social group composition; social groups are determined by individuals sleeping in the same burrow. We used t‐tests to compare the number of breeding females per group and the number of females and males per hectare, calculated as the total number of squirrels in the study site divided by the size of the study area in hectares. Adult males either disperse at reproductive maturity or delay dispersal and remain with their natal group (O'Brien et al., 2021; Waterman, 1995, 1997). Both dispersal tactics result in reproductive success (Manjerovic & Waterman, 2015); thus all adult males present were included, regardless of dispersal tactic, in our calculations for the number of males per hectare. Site comparisons were analyzed using a t‐test.

### 2.4 Mating behaviors

Between 2002 and 2006, we collected detailed behavioral data on days of estrus following methods established for this species at the low resource site during 1989–1991 (Waterman, 1995, 1997, 1998). We could tell estrus was imminent by increased activity by males towards the female coming into estrus (Waterman, 1997). We recorded all interactions and copulations; because copulations occur both above and below ground, we assumed copulation occurred below ground if a female was followed into a burrow by a male and they remained underground for at least 1 min (Waterman, 1998). Estruses generally lasted approximately 3 h and were considered over when females left the area, rejected males, or if males stopped searching and started feeding (Waterman, 1998). After every estrous event, we immediately put out traps to capture the estrous female to look for evidence of copulatory plugs. We were unable to record data blind because we used only focal field animals. Methods used to gather mating behavior data did not differ between sites or study years.

Based on estrous events, we determined the mean duration of estrus and number of mates per female, and the mean copulatory success for males. Estrous events included individual females that were in the data set over multiple years. We accounted for multiple occurrences of the same female by using a generalized linear mixed model (GLMM) with a Poisson‐distributed error, including female ID as a random variable and site as a predictor factor. This model also is commonly used if dependent variables violate normality assumptions (Agresti, 2002). Given that generally a single female was in estrus in an area at any one time, we calculated the operational sex ratio as the number of sexually active males present during each estrus. We used this metric to compare sexual selection intensity (Emlen & Oring, 1977; Waterman, 1998) but also calculated the opportunity for sexual selection at each site across the study period as the variance in copulatory success per individual divided by the squared mean of success (*I*_*s*_ = SD^2^/mean^2^; Shuster & Wade, 2003; Wade & Arnold, 1980). Although research has suggested that the “Jones index” (*s*'_max_; Jones, 2009) outperforms the former by measuring how mating success varies with reproductive success (Henshaw et al., 2016), we were unable to calculate a direct correlation between mating and reproductive success, given limited mating observations and high estrus failures (Waterman, 1996).

### 2.5 Male investment

Because dominance has been demonstrated to influence the male reproductive success (Waterman, 1998), we recorded all male–male approach–displacement interactions in both populations from 2002 to 2006 to calculate dominance relationships using Landau's index of linearity (Lehner, 1998; Waterman, 1998). We also recorded any evidence of copulatory competition including copulatory calls or mate guarding (Sherman, 1989), and compared sites using a chi‐square test.

To assess male movement patterns and thus access to females, we radio‐collared 16 males at our high resource site in 2006 (Model SOM‐2380; Wildlife Materials, Inc.) but removed two males from analysis due to fewer than 50 locations. After collaring, we released all animals at the site of capture and waited at least 24 h before locating animals to allow time for acclimation. Between May and July 2006, we located animals a minimum of 50 times, split between day and night, and also included locations based on observations and trapping. Although other methods are widely used (Horne & Garton, 2006), we generated 95% MCP for comparison to the low resource site, which was calculated previously using the same methods from 1989 to 1991 (Waterman, 1995). We used ArcMap v.9.3.1 (ESRI) and the animal‐movement extension (Beyer, 2004) to estimate male home ranges.

To assess reproductive investment, we measured internal male reproductive morphology in a subset of adult males in 2006. We euthanized animals on site with either halothane or chloroform overdose based on availability and country permit requirements (see Manjerovic et al., 2008). We used electronic calipers (Mitutoyo Inc.) to measure scrotal width and length to the nearest ±1.0 mm, which included both the epididymis and testis. We measured the mass (±0.1 g) of the testes and bulbourethral gland and calculated relative testes size following Kenagy and Trombulak (1986). We corrected measurements for body size before comparing between sites.

### 2.6 Reproductive success

We extracted total genomic DNA from all captures between 2002 and 2006 using a DNeasy Kit (Qiagen Inc.) and genotyped all individuals using eight species‐specific microsatellite loci (Manjerovic et al., 2009). Primer sequences are available on GenBank (accession nos. FJ823123‐FJ823131), and polymerase chain reaction conditions and cycling parameters are described in Manjerovic et al. (2009). We amplified PCR products on a Beckman 8000 CEQ and used corresponding software to size alleles compared with internal standards. We tested for Hardy–Weinberg deviations and linkage disequilibrium using Genepop with *α* = .05 (Raymond & Rousset, 1995). To determine limits for individual identification, we used the program GIMLET v 1.3.3 (Valière, 2002) to calculate the probability that two individuals randomly show identical genotypes (P_ID,_ Paetkau & Strobeck, 1994) and the P_ID_ among siblings (P_IDsib_; Evett & Weir, 1998). To quantify male reproductive success, we assigned the parentage of all juveniles and subadults using a likelihood‐based approach in CERVUS v.3.0 (Kalinowski et al., 2007; Marshall et al., 1998). This program assigns paternity based on confidence levels calculated using simulated data that includes population allele frequencies, proportion of population sampled and genotyped, and error rates. We ran a total of 100,000 iterations using a 1% genotyping error rate (Manjerovic & Waterman, 2015). We calculated a mean of four adult female candidates per social group and 11 candidate males based on the operational sex ratio at the time of breeding (Waterman, 1998). We calculated the proportion of sampled candidates as 10% for females and 30% for males by subtracting the proportion of unknown adults captured each subsequent year. Regardless of whether or not males were recaptured, we included all adult males as candidates in each subsequent year of their initial capture. Accounting for this variation in sampling effort along with changes in reproductive ages of males each year resulted in different candidate male groups each year.

We calculated maternity and paternity using individuals typed at a minimum of 6 loci with at least an 80% confidence level (Wells et al., 2017). We did not exclude parents based on 1 mismatch with offspring, allowing identification of the most likely parent from among multiple nonexcluded parents (Kalinowski et al., 2007). For maximum reliability of paternity assignments, we only included juveniles with a 95% confidence in the assigned mother, and we only accepted parentage assignments when there was no more than 1 mismatch for assumed mother–father‐offspring relationships. The total exclusionary power for the data set was over 98% (Jamieson & Taylor, 1997). We used genetic paternity assignments (i.e., numbers of sired offspring) to calculate variance in male reproductive success (*V*_males_) between sites and included all adult males present regardless of whether they sired any offspring. We used variance in male reproductive success divided by the squared mean of male reproductive success to calculate the opportunity for sexual selection (*I*_males_) (DuVal & Kempenaers, 2008; Shuster & Wade, 2003).

We tested that all data were normal and homoscedastic; data that did not meet those assumptions were either log‐transformed, or tested using nonparametric statistics or models suitable for non‐normal response variables. All data were tested for significance either in JMP v.8 (SAS Institute Inc.) or RStudio (v1.1.463) and considered statistically significant at *α* = .05. Results are expressed as mean ± SE unless otherwise stated.

## 3 RESULTS

### 3.1 Site variation

**FIGURE 2:**
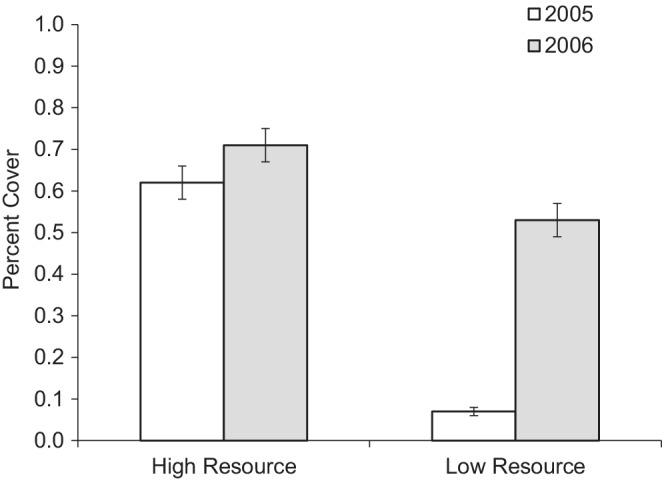
Differences in vegetation percent cover (mean ± SE) support South Africa as our high resource site and Namibia as our low resource site.

**FIGURE 3:**
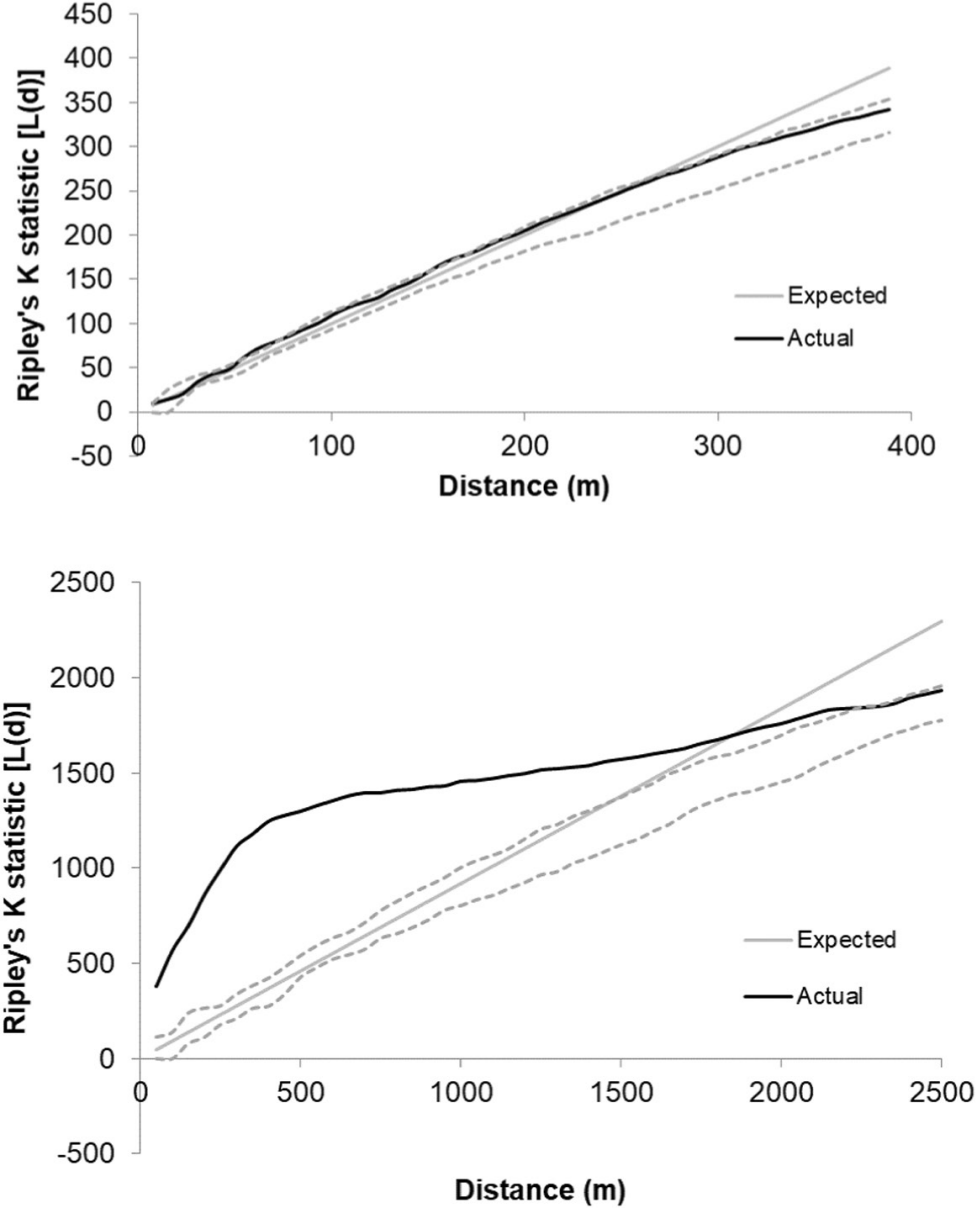
Distance from the center of each *Xerus inauris* burrow (m) versus modified Ripley's K (L‐Distance function) in our high resource South African site (top) and our low resource Namibian site (bottom) measured in 2006. Expected line (light gray) indicates complete spatial randomness; gray dashed lines represent upper and lower confidence estimates. Actual patterns that fall above the confidence interval represent statistically significant clustering while lines that fall below represent statistically significant dispersion.

We found statistically significant differences in vegetation between burrow clusters in our high and low resource sites (two‐way anova: *F*_1,12_ = 33.3, *n* = 6, *p* < .01). As expected, our low resource site had less vegetation and greater variability among years differing by an order of magnitude from our high resource site and between years (Figure 2). We found unequal variance of annual rainfall between sites (Levene test: *F*_1,52_ = 6.45, *n* = 27 years, *p* = .01) with over twice as much yearly rainfall during the current study in the high resource site (mean: 546.4 mm ± 57.9) compared with the low resource site (mean: 238.9 mm ± 64.4; *t* test: *t*_4_ = 7.9, *p* < .01). Using spatial analysis, we found the low resource site had a lower density of burrow clusters (0.26/ha) and statistically significant clustering across greater distances (0–1500 m) compared with the high resource site where there was a higher density of burrows (8.41/ha) that were randomly distributed at distances <500 m (Figure 3). Consequently, mean distances (M ± SE) between burrow clusters in the high resource site were significantly shorter (154.12 ± 9.2, *n* = 9) compared with the low resource site (248.04 ± 27.2, *n* = 7; *t* test: *t*_14_ = −3.62, *p* < .01).

### 3.2 Demographics

From 2002 to 2006, we found no statistically detectable differences between the number of breeding females per social group (high resource: mean: 3.7 ± 0.4, range: 2–8, *n* = 10; low resource: mean: 4.6 ± 0.5, range: 2–10, *n* = 7; *t* test: *t*_15_ = −1.48, *p* = .08). However, the yearly number of adult females per hectare was an order of magnitude greater in the high resource site (6.1 ± 0.75) compared with the low resource site (0.22 ± 0.05; *t* test: *t*_4_ = 7.74, *p* < .01). Over the same 5 years, adult males per hectare were also greater in our high resource site (6.60 ± 0.95) compared with our low resource site (0.24 ± 0.05; *t* test: *t*_4_ = 6.70, *p* < .01).

### 3.3 Mating behaviors

We recorded a total of 38 estrous events at the high resource site from 2002 to 2006. However, so few estrous events were observed in our low resource site during the current study (*n* = 4) that we included estrus data collected from this same site prior to this study (*n* = 31, 1989–1990) (Waterman, 1998). These data were collected using the same methods and we found rainfall and density of adult males and adult females per hectare to be similar between time periods (Table 1). In addition, female groups lived in the same burrow clusters during both time periods; thus, we felt confident using these data in our final comparison. We found estruses were longer in the low resource site; however, this pattern was driven by a single outlier (600 min; mean: 197.77 ± 6.5 min) that, when removed, resulted in no differences in estrus duration (GLMM: *t* = −1.93, *p* = .06; Table 2). The intensity of male–male competition, as measured by the operational sex ratio, did not differ between sites (GLMM: *t* = −0.84, *p* = .41; Table 2). We also found similar opportunities for sexual selection at both sites as measured by variance in copulatory success divided by the squared mean of success (high resource *I*_*s*_ = 2.9; low resource *I*_*s*_ = 2.7). Despite these similarities, we found female *X. inauris* from the low resource site averaged three more copulations per estrus compared with females in the high resource site (GLMM: *t* = −1.99, *p* = .05; Table 2). Consequently, male copulatory success was greater in the low resource site where males averaged 0.75 copulations per individual per estrus compared with 0.47 copulations per individual per estrus in the high resource site. We also found a higher number of unknown males in our high resource site with an average of at least 1 unmarked male attending each estrus at this site (GLM: *t* = 2.19, *p* = .03; Table 2).

**TABLE 1:** Comparison of annual rainfall and adult squirrel density at the low resource site from 1989 to 1991 (*n* = 3) and the current study from 2002 to 2006 (*n* = 5)

	1989–1991	2002–2006	*t*_6_	*p*
	Mean ± SE	Mean ± SE		
Rainfall	272.00 ± 12.9	225.54 ± 53.4	0.65	.54
Male density	0.40 ± 0.03	0.34 ± 0.06	0.77	.47
Female density	0.39 ± 0.06	0.48 ± 0.07	−0.95	.38

**TABLE 2:** Comparison of *Xerus inauris* mating behaviors between a high resource site (South Africa) and a low resource site (Namibia) collected between 2002 and 2006

Mating variables	High resource (South Africa)			Low resource (Namibia)			*t*	*p*
	*N*	Mean ± SE	Range	*N*	Mean ± SE	Range		
Estrus duration (min)	38	163.53 ± 8.8	70–290	34	188.21 ± 15.5	40–375	−1.93	.06
Operational sex ratio	38	10.84 ± 0.6	5–19	34	11.12 ± 0.6	3–18	−0.84	.41
Number of mates/estrus	33	3.24 ± 0.3	1–6	34	4.06 ± 0.4	1–8	−1.80	.08
Number of copulations/estrus^a^	33	5.00 ± 0.6	1–13	34	7.62 ± 1.1	1–24	−1.99	.05
Number of unknown males present	38	1.47 ± 0.3	0–8	34	0.92 ± 0.2	0–3	2.19	.03

*Note*: Low resource site data also include data collected during a previous study from 1989 to 1991 (Waterman, 1995, 1998). Operational sex ratio is measured as the number of males present and attentive to an estrous female.

^a^ log‐transformed for analysis; raw data presented.

### 3.4 Male investment

We found no evidence of a dominance hierarchy among males in the high resource site between 2002 and 2006 with a mean linearity index of 0.08 ± 0.05 (range: 0.04–0.18). However, mate guarding was more likely to occur in this site compared with the low resource site (chi‐square test: χ^2^ = 5.87, *p* = .02) with males guarding females in 26% (10/38) of estruses compared with 6% (2/34) in the low resource site. In 2006, we recorded a mean of 104 locations per male (range: 59–166) resulting in a mean home range of 21.6 ± 4.2 ha (range: 6.7–61.0), which was almost twice that of males from the low resource site (12.5 ± 2.5 ha; Waterman, 1995). We found no differences in male body mass between the two sites in 2005 and 2006, but males in our high resource site had testes, epididymides, and bulbourethral glands that were approximately 30% larger than males in the low resource site (Table 3).

**TABLE 3:** Comparison of *Xerus inauris* male morphology between high resource (South Africa) and low resource (Namibia) populations

	High resource (South Africa)				Low resource (Namibia)				df	*p*
Morphological character	*N*	Mean ± SE	Range	CV	*N*	Mean ± SE	Range	CV		
Body mass (g)	31	667 ± 8.7	575–800	7.3	25	657.2 ± 15.2	515–805	11.5	54	.396
Testes mass (g)	29	12.5 ± 0.3	8.3–16.6	12.5	25	9.0 ± 0.5	4.5–14.0	24.8	52	<.001
Epididymal mass (g)	29	6.8 ± 0.3	4.7–9.8	23.9	24	4.7 ± 0.3	2.4–6.4	26.3	51	<.001
Bulbourethral gland mass (g)	22	7.7 ± 0.4	3.8–11.5	27.2	14	5.8 ± 1.1	1.2–13.6	68.9	34	.032
Relative testes size^a^	29	2.7 ± 0.1	1.9–3.2	11.5	25	2.0 ± 0.1	1.0–2.9	25.3	52	<.001

*Note*: Data were collected between 2005 and 2006. Mean values and ranges for morphometrics indicate actual values before correcting for body size; statistics run on values corrected for body size.

^a^ As calculated in Kenagy and Trombulak (1986).

### 3.5 Reproductive success

**FIGURE 4:**
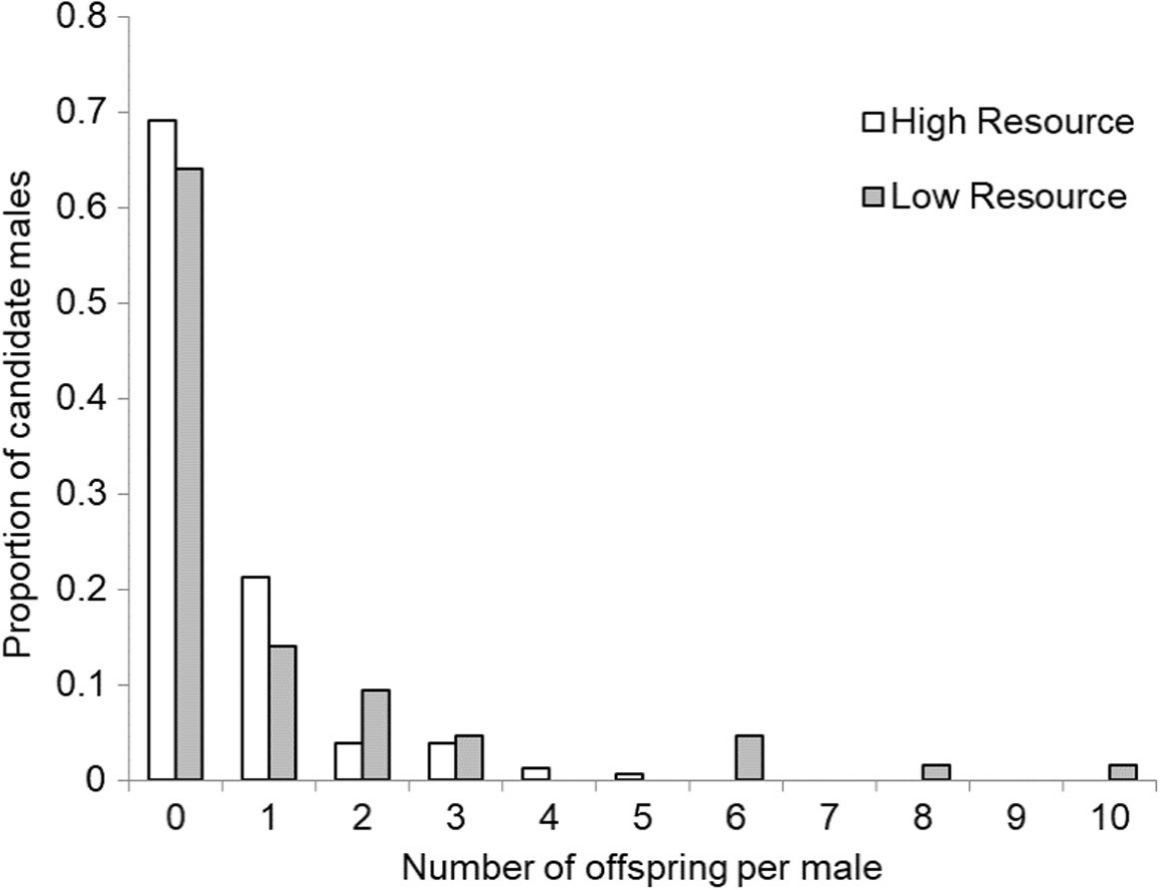
Reproductive skew among male *Xerus inauris* at two sites in southern Africa based on genetic paternity assignments from data collected between 2002 and 2006.

Between 2002 and 2006, we genotyped 387 individuals from the high resource site and 322 individuals from the low resource site. We found no deviations from Hardy–Weinberg equilibrium or evidence of linkage disequilibrium after Bonferroni correction (Table S1). We assigned paternity to 76 of 155 juveniles from our high resource site (49%) and 66 of 102 juveniles from our low resource site (65%) (Table 4). Reproduction was extremely skewed among individuals (Figure 4), but the distribution of offspring among males was not statistically different between sites (Wilcoxon Ranked Sums: *Z* = 1.41; *p* = .159). Approximately 65% of the males at both sites sired no offspring (69.0% high resource, 64.1% low resource) resulting in extremely high and similar intensities of sexual selection (high resource *I*_males_ = 4.8; low resource *I*_males_ = 3.5). Out of those males that did successfully sire offspring, the majority from the high resource site sired a single offspring (33/48) while the majority sired multiple offspring from the low resource site (14/23). This skew resulted in more individuals from the low resource site siring multiple offspring and a higher variance in fertilization success (*V*_m_ = 4.20) compared with the high resource site (*V*_m_ = 0.85). We did find evidence of multiple paternity in 62.5% of sibship pairs from our high resource site (*n* = 8) and 61.5% from the low resource site (*n* = 13) when both offspring were assigned fathers.

**TABLE 4:** Characteristics of juvenile paternity assignments of *Xerus inauris* in two study sites between 2002 and 2006

	High resource (South Africa)					Low resource (Namibia)				
Year	Candidate males	No. of juveniles	No. of assigned juveniles (%)	No. of sires	% of male sires	Candidate males	No. of juveniles	No. of assigned juveniles (%)	No. of sires	% of male sires
2002	39	23	9 (39)	7	18	33	12	8 (67)	6	18
2003	54	19	10 (53)	9	17	21	2	2 (100)	2	10
2004	78	57	19 (33)	17	22	23	57	36 (63)	11	48
2005	60	42	33 (79)	21	35	32	13	12 (92)	7	22
2006	97	56	24 (43)	21	22	38	32	18 (56)	10	26

## 4 DISCUSSION

We found male *X. inauris* vary both in reproductive behavior and morphology between populations that differ in resource availability. Our site with higher rainfall had significantly higher percent cover and less variability in percent cover between years compared with our low resource site, supporting a link between rainfall and primary productivity (Happold & Happold, 1992; LaFlèche & Waterman, 2020). In arid environments, population densities can fluctuate widely with changes in rainfall altering social group dynamics (Waterman, 2002). For example, in striped mice (*Rhabdomys pumilio*), another rodent species from southern Africa, intraspecific variation in their social system is impacted by both resources and population density. Mice tend to remain solitary in populations with greater rainfall and in years when population density is low but form groups in areas of lower rainfall or when population density is high (Schradin et al., 2010; Schradin et al., 2020; Schradin & Pillay, 2005). We found differences in density between sites with more adults per hectare in our high resource site but little variability from year to year within sites. We also found differences in suitable habitats with a greater density of burrow clusters spread out more evenly across the landscape in our high resource site. Burrows in our low resource site clustered together, resulting in a patchy distribution across the landscape. In great gerbils (*Rhombomys opimus*), another arid‐adapted ground‐dwelling species, significant clustering of occupied burrows was attributed to a combination of landscape and ecological factors influencing habitat suitability and dispersal behaviors (Wilschut et al., 2015). Female *X. inauris* tend to demonstrate strong site fidelity while males are more nomadic, moving around in search of females. Despite these differences, both males and females depend on these burrow systems daily and such differences in the distribution of burrow clusters between sites may affect the frequency that males and females associate with one another.

Males in our high resource site had an average home range that was almost twice that of males in our low resource site (Waterman, 1995) and therefore encountered more adult females. When resources influence mate availability in both time and space, different reproductive strategies often emerge (Shuster & Wade, 2003). In oribi (*Ourebia ourebi*), females form larger groups and smaller home ranges during periods of abundant grasses. Male oribi responds to these differences in female distribution and behavior by altering precopulatory mating behaviors, maintaining active territories when females were clustered and defending females when they ranged widely (Brashares & Arcese, 2002). In tropical ground squirrels, reproduction often is restricted by the seasonal rainy season or unpredictable periods of resource scarcity, while North American ground squirrels are constrained by short, discrete breeding seasons that last only a few weeks after females emerge from hibernation (Waterman, 1996). These highly synchronous breeding seasons result in multiple receptive females in a single day, such that males often leave to search and compete for additional females after the initial mating bout to gain greater reproductive advantages (Lacey & Wieczorek, 2001; Raveh et al., 2010; Sherman, 1989). Elongating the breeding season or distance between receptive females affects the costs and benefits of acquiring partners and alters which mating strategies are most successful (Brashares & Arcese, 2002; Schwanz et al., 2016; Shuster & Wade, 2003). *X. inauris* differ from other sciurids as they are not constrained by winters and can breed year‐round (Waterman, 1996). There appears to be no predictability to female receptivity as multiple females rarely come into estrus on the same day and are asynchronous breeders with spontaneous ovulation (Bouchie et al., 2006; Waterman, 1996). Because receptive females are scattered more evenly across a greater distance in areas of higher resources, waiting for receptive females may be less advantageous if it reduces the likelihood of gaining additional breeding opportunities.

Males detect the onset of estrus and aggregate around the female during the days leading up to her estrus, associating regularly with one another (Waterman, 1997; Waterman, 1998). Only males in the low resource site form dominance hierarchies (average yearly Landau h > 0.9, Waterman, 1995, compared with average yearly h < 0.2 in the high resource site, this study) suggesting males may respond to the clustered distribution of females. The low resource site appears to represent a more closed system, where females are clustered together and dominance hierarchies are easily maintained. However, the high resource site is more open (van der Marel et al., 2020), averaging at least one unknown male during each estrus, such that inconsistency in male–male interactions may reduce the likelihood of forming stable dominance hierarchies. Males in these sites may offset a mating advantage determined by dominance with increased investment in postcopulatory competition. Larger testes, epididymis, and bulbourethral glands suggest increased investment in sperm competition through ejaculate investments and by discharging copulatory plugs (Ramm et al., 2005). We frequently recovered copulatory plugs from high resource females but never from low resource females, although we cannot rule out the possibility of female removal of copulatory plugs as documented in tree squirrels (Koprowski, 1992).

Postcopulatory mechanisms often are an attempt to deter other males from mating to minimize sperm competition. Guarding was significantly more likely to occur in our high resource site after mating and is often seen when receptive females are further apart such that locating additional mating opportunities is time‐consuming (Sherman, 1989). These conditions may lead to a last male advantage, as seen in Idaho ground squirrels (*Spermophilus brunneus*), where unguarded females mate with additional males and the last guarding male sires most of the offspring (Sherman, 1989). By contrast, male Belding's ground squirrels (*S. beldingi*) do not range as widely as for females, and males are more likely to resume mate searching after copulation (Sherman, 1989). We observed several occurrences of postcopulatory calls in our high resource site but never in the low resource site. In black‐tailed prairie dogs (*Cynomys ludovicianus*), mating calls are given both before and after copulating and are thought to be directed at both sexes. Although the first copulating male was significantly more likely to call, these calls did not deter other males (Grady & Hoogland, 1986). In other species, like the fallow deer (*Dama dama*), calls are an intrasexual threat directed at competitors (McElligott & Hayden, 2001). Given the low probability for multiple *X. inauris* females to come into estrus on the same day (Waterman, 1996) and the differences in density and distance between sites, these postcopulatory mechanisms may be a response to increased sperm competition while not imposing a cost to males in terms of a fitness tradeoff.

Differences in competitive strategies, such as territoriality or dominance, often result in extreme variance in male fertilization success, where a small number of males are responsible for the majority of matings (DuVal & Kempenaers, 2008). Approximately two‐thirds of males at both sites never sired an offspring among sampled juveniles. We did find a higher variance in fertilization success with fewer individuals siring the majority of the offspring in our low resource site where dominant males obtain a greater proportion of copulations (Waterman, 1998). Of the successful males, 60% of males sired more than 1 offspring in our low resource site compared with 30% in our high resource site. Such uneven distribution of reproductive success among males is not surprising given that *X. inauris* have asynchronous, short periods of female receptivity (Shuster & Wade, 2003; Waterman, 1998) and consequently one of the highest operational sex ratios among sciurids (11 M:1F; range 3–18; Waterman, 1997; Waterman, 1998). We found less variance in copulatory success among individuals compared with fertilization success at both sites. In our species, over 70% of all breeding events fail to wean offspring and therefore there is a low likelihood of paternity for each male attending an estrus (Pettitt et al., 2008; Waterman, 1996). Low resources can alter conditions for mating and the opportunity for sexual selection, especially in arid‐adapted species. In Iberian red deer (*Cervus elaphus hispanicus*), reproductive timing and behavior are closely tied to rainfall patterns (Millán et al., 2021). When environmental conditions are poor (i.e., low rainfall), males delayed rutting and decreased rutting intensity in response to females. This change in turn favored a higher degree of polygyny and increased opportunity for sexual selection (Millán et al., 2021). While resource availability does not appear to influence female reproductive output in *X. inauris* (Pettitt et al., 2008) or the number of breeding females per social group, male reproductive output did differ between sites. Males at both sites had estimates of sexual selection intensity similar to lekking species, with mating extremely skewed towards specific individuals within the populations.

Resources play a pivotal role in physiological tradeoffs between reproductive and behavioral strategies especially when body condition and/or the maintenance of secondary sexual characteristics affect male reproduction. When female oribi are dispersed over a larger area, males spend more time and energy traveling greater distances and consequently spend less time resting compared with males that maintained territories (Brashares & Arcese, 2002). In striped mice, females who maintain smaller home ranges also experience delayed reproduction and dispersal (Schradin & Pillay, 2005). While increased investment in reproductive anatomy may be attributed to higher resource availability, male *X. inauris* also have different dispersal tactics that are impacted by rainfall (O'Brien et al., 2021). Sexually mature males either disperse at reproductive maturity and join a male band (Waterman, 1995), or delay dispersal and remain with their natal group (O'Brien et al., 2021; Waterman, 1995; Waterman, 1997). Dispersal tactics result in similar reproductive success (Manjerovic & Waterman, 2015), but physiological and behavioral differences are affected by resources (O'Brien et al., 2021; Scantlebury et al., 2008). Band males are more mobile, with higher resting metabolic rates and larger home ranges and consequently spend less time feeding (Manjerovic & Waterman, 2015; Scantlebury et al., 2008). Despite these higher costs, band males are only in better body condition during periods of high resources. During low rainfall years, body condition, physiological indices, and ectoparasite loads indicate band males have poorer body conditions (O'Brien et al., 2021). Resources likely play an important role in impacting male dispersal tactics (Scantlebury et al., 2008), but the role of resources in reproductive success across the sites is an area that needs further exploration.

Reproductive success is a product of both copulation and fertilization; thus, there are multiple opportunities for selection to affect reproductive morphology and behavior. Previous research determined resource availability does not influence female reproductive output (Pettitt et al., 2008), but resources do impact female distribution altering male competitive strategies. Male *X. inauris* at both sites lacked overt competition, but successful reproductive strategies differed between sites as males responded to female distribution and availability. Differences in burrow clusters and female density could impact male home ranges and the frequency of males encountering both estrous females and male competitors. Higher rainfall and primary productivity resulted in greater and more predictable resources, which likely affects male reproductive investment. These factors, combined with differences in burrow distribution across the landscape, may alter costs in competitive searching behaviors among males. Competitive searching occurs in both populations, but dominance was an important determinant of success in sites where resources are limited, whereas sperm competition was more pronounced in areas with more abundant resources. Our study provides a deeper understanding of how environmental factors influence both male pre‐ and postcopulatory strategies in a species where mating success does not rely on male–male aggression.

## AUTHOR CONTRIBUTIONS

**Mary Beth Manjerovic:** Conceptualization (equal); data curation (equal); formal analysis (lead); investigation (equal); methodology (lead); writing – original draft (lead); writing – review and editing (lead). **Eric A. Hoffman:** Formal analysis (supporting); methodology (supporting); writing – original draft (supporting); writing – review and editing (supporting). **Christopher L. Parkinson:** Formal analysis (supporting); methodology (supporting); writing – original draft (supporting); writing – review and editing (supporting). **Jane M. Waterman:** Conceptualization (equal); data curation (equal); formal analysis (supporting); funding acquisition (lead); investigation (equal); methodology (equal); project administration (lead); writing – original draft (supporting); writing – review and editing (supporting).

## CONFLICT OF INTEREST

The authors state that there is no conflict of interest.

## Supporting information


Table S1

## Data Availability

The data that support the findings of this study are available from the corresponding author upon reasonable request. All data are associated with tables and figures: Dryad https://doi.org/10.5061/dryad.jsxksn0cv.
