# Supplementary material for: Intraspecific variation in male mating strategies in an African ground squirrel (Xerus inauris)
Source: Ecol Evol. 2022 Aug 15;12(8):e9208. doi: 10.1002/ece3.9208 (PMC9379349; doi:10.1002/ece3.9208)
Supplement: Supplementary file 1 — Table S1 [file ECE3-12-e9208-s001.docx]

**Supplemental Data**

**Appendix A:** Summary of genetic diversity by populations: number of alleles (Na), expected H_E_ and observed heterozygosity H_O_, and probability of identification of individuals (P_ID_) and of siblings (P_IDsib_)

| Locus | | Na | H_E_ | H_O_ | P_ID_ | P_ID(sib)_ |
| --- | --- | --- | --- | --- | --- | --- |
| South Africa | |  |  |  |  |  |
|  | Xin1 | 7 | 0.7 | 0.62 | 1.50E-01 | 4.38E-01 |
|  | Xin8 | 7 | 0.64 | 0.64 | 1.79E-01 | 4.76E-01 |
|  | Xin9 | 9 | 0.6 | 0.58 | 1.99E-01 | 4.98E-01 |
|  | Xin12 | 4 | 0.59 | 0.55 | 2.59E-01 | 5.22E-01 |
|  | Xin10 | 8 | 0.75 | 0.65 | 9.84E-02 | 3.99E-01 |
|  | Xin4 | 4 | 0.6 | 0.51 | 2.38E-01 | 5.08E-01 |
|  | Xin5 | 4 | 0.64 | 0.58 | 2.03E-01 | 4.82E-01 |
|  | Xin3 | 9 | 0.69 | 0.65 | 1.45E-01 | 4.40E-01 |
|  | |  |  |  |  |  |
| Namibia | |  |  |  |  |  |
|  | Xin1 | 11 | 0.74 | 0.7 | 1.08E-01 | 4.09E-01 |
|  | Xin8 | 10 | 0.69 | 0.68 | 1.26E-01 | 4.35E-01 |
|  | Xin9 | 7 | 0.73 | 0.67 | 1.21E-01 | 4.16E-01 |
|  | Xin12 | 5 | 0.47 | 0.48 | 3.36E-01 | 5.97E-01 |
|  | Xin10 | 10 | 0.72 | 0.55 | 1.11E-01 | 4.16E-01 |
|  | Xin4 | 11 | 0.7 | 0.44 | 1.22E-01 | 4.33E-01 |
|  | Xin5 | 19 | 0.86 | 0.79 | 3.22E-02 | 3.30E-01 |
|  | Xin3 | 12 | 0.69 | 0.62 | 1.45E-01 | 4.39E-01 |
